# Monitoring the Mental Health and Professional Overload of Health Workers in Brazil: A Longitudinal Study Considering the First Wave of the COVID-19 Pandemic

**DOI:** 10.3389/fpsyt.2022.852157

**Published:** 2022-04-08

**Authors:** Flávia L. Osório, Antonio Waldo Zuardi, Isabella L. M. Silveira, José Alexandre S. Crippa, Jaime Eduardo Cecílio Hallak, Karina Pereira-Lima, Sonia R. Loureiro

**Affiliations:** ^1^Medical School of Ribeirão Preto, University of São Paulo, São Paulo, Brazil; ^2^Department of Psychiatry, Federal University of São Paulo, São Paulo, Brazil

**Keywords:** anxiety, mental health, follow-up, COVID-19, healthcare personnel, burnout, psychological

## Abstract

Few longitudinal studies assessed the less immediate consequences of the COVID-19 pandemic on health workers' mental health, especially in less developed countries. The objective was to assess the evolution of mental health indicators of Brazilian health workers providing care to COVID-19 patients, considering the beginning and first wave of the pandemic, identifying risk and protective factors. A non-probabilistic sample of health professionals was assessed for 6 months at seven different points in time using standardized instruments to measure anxiety, depression, insomnia, posttraumatic stress, and burnout symptoms. Risk and protective factors were assessed using a questionnaire addressing socio-demographic, clinical, occupational variables, and COVID-19 risk perception. The results indicate high rates for all the indicators (>30%) throughout the follow-up; only anxiety symptoms decreased in the different phases compared to the baseline. Depression and insomnia symptoms showed a significant drop in isolated points of the assessment, which were not maintained at the final follow-up. Burnout indicators concerning emotional exhaustion and depersonalization remained stable (40 and 20%), while professional achievement decreased by approximately 19%. Occupational and personal characteristics (profession and work setting), perceptions regarding protective measures imposed by the institutions, and future professional prospects stood out as risk/protective factors in mental health. Unlike European and Asian countries, where mental distress symptoms tended to decrease over the pandemic, this study's results suggest alarming indicators of mental health problems remaining stable with burnout symptoms on the rise. Hence, the different contexts across countries, with different management resources and investments in health actions, seem to influence workers' mental health differently, demanding constant attention and monitoring and measures to minimize the impacts on individuals and collectives, especially in less developed countries like Brazil.

## Introduction

Health workers are considered vulnerable to mental health problems within the COVID-19 pandemic due to their intense exposure to multiple stressors, suggesting a need for public health policies intended to favor personal conditions and the quality of care delivery (1).

The prevalence of mental health problems among health workers is highly reported in studies conducted in various countries, in different times of the COVID-19 pandemic (2), especially anxiety, depression, insomnia, posttraumatic stress, and burnout, in addition to other general concerns with one's health and fear of infection (3).

Previous studies conducted in the last 20 years in epidemic and pandemic contexts worldwide, including the COVID-19, report various risk conditions leading to the development of mental symptoms and disorders among health workers, mainly: being a woman, working in the nursing field and the frontline, longer shifts, having inappropriate personal protective equipment, having insufficient knowledge regarding the virus, inappropriate training, fewer years of professional experience, and lack of social support (4).

Considering the current pandemic, Osório et al. (5) identified that occupational variables stand out as risk factors for different groups of professionals providing care to individuals with COVID-19 in Brazil. Despite the recognition that multiple conditions represent risk factors for the exacerbation of mental health problems, in low- and middle-income countries, such as Brazil, the scarcity of health system resources exerts additional pressure, associated with the lack of basic equipment and treatment resources (6, 7), making fighting the pandemic even more challenging.

In addition, multiple conditions represent risk factors that compound mental health problems in low- and middle-income countries, such as Brazil, and the scarcity of health system resources exerts extra pressure, associated with a lack of basic equipment and care resources (6, 7) so that fighting the pandemic is even more challenging.

There are few longitudinal studies thus far assessing the less immediate consequences of the pandemic on the health workers' mental health. Most studies assessed the initial impact of the pandemic and specific aspects of its evolution, which, from an epidemiological perspective, is constantly changing worldwide, though it remains persistent with times in which the pandemic peaks and then subsides (8, 9). The few longitudinal studies available are concentrated in European and Asian countries, which restrict the generalization of studies, given social and economic specificities, especially compared to Latin American and African countries; thus, studies addressing these contexts are needed. To the best of our knowledge, only one longitudinal study was conducted in Latin America (10).

Therefore, this study's primary objective was to assess the evolution of mental health indicators of Brazilian health workers providing care to COVID-19 patients, considering the beginning and first wave of the pandemic, identifying risk and protective factors.

## Method

This longitudinal study, called MENTALvid, included a non-probabilistic sample composed of Brazilian health professionals from different fields, responsible for providing care to COVID-19 patients during the beginning and first wave of the pandemic, including physicians (regardless of the specialty), nursing workers (nurses, nursing technicians/aids, and radiology technicians), and other professionals (bachelor's degree holders working in the hospital setting: psychologists, physical therapists, speech therapists, occupational therapists, dentists, pharmacists, and social workers). The participants were recruited on social media (e.g., Facebook, Instagram, WhatsApp), traditional media (TV and radio), and by contacting class councils and health organizations in the various Brazilian regions. Participation in the study was voluntary and required signing a free and informed consent form. All the participants who completed the instruments at the baseline were included. The study was submitted to and approved by the Institutional Review Board (Process 4.032.190).

Data collection was initiated on May 19th (baseline) and lasted until August 23rd, 2020, when the mark of 1,500 individuals (expected sample) was obtained according to criteria proposed by the Chinese pioneer study (11). At the beginning of the study, the first COVID-19 case had been officially diagnosed in Brazil 82 days ago. The number of confirmed cases was 271,628 and 17,971 deaths, with peaks in various Brazilian regions. The follow-up (D90) ended on November 21st, 2020, with 6,052,786 cases and 168,989 deaths. The daily growth rates of new cases in the months when data were collected were: May 6.2%, June 3.3%, July 1.4%, August 1%, September 0.7%, October 0.4%, November 0.5% (12).

### Instruments

(a) To characterize the sample and assess protective and risk factors in mental health:
  - A questionnaire was developed to characterize socio-demographic and occupational factors and identify risk perception of COVID-19. The instrument is composed of 39 questions addressing numerical variables such as age and years of professional experience, and categorical variables including sex, marital status, whether the individual lives alone or with a partner and/or children, has a religion (yes/no), smoke, use drugs, consume alcohol (yes/no), regular exercise (yes/no), previous psychiatric care and psychiatric diagnosis (yes/no), physical illness/medication use (yes/no), profession (nurse/physician, others), workplace (public/private hospital), type of care facility (secondary/tertiary care) and whether it is a referral center for COVID-19 (yes/no), frontline (yes/no), extra working hours (yes/no), desire to quit job (never-rarely/often-always), positive professional prospects (yes/no), whether is satisfied with the physical protective measures adopted by the facility (never-rarely/often-always), receives any support from the institution (yes/no), social/emotional support from coworkers (yes/no), was infected by the Sar-CoV-2 (yes/no), is concerned with being infected or infect family members with the Sar-CoV-2 (yes/no), notices that people avoid social contact because of the profession (yes/no).
(b) To assess outcomes:
  - Generalized Anxiety Disorder-7 (GAD-7): a 7-item self-report instrument that screens anxiety-associated symptoms rated on a three-point scale ranging from 0 (never) to 3 (almost every day). It was proposed by Spitzer et al. (13) and validated in Brazil by Moreno et al. (14). A cutoff score ≥ 10 corresponds to 89% of sensitivity and 82% of specificity;
  - Patient Health Questionnaire-9 (PHQ-9): a 9-item self-report instrument intended to assess depression indicators. It was proposed by Kroenke et al. (15) and validated in Brazil by Osório et al. (16). Its items are rated from 0 (“never”) to 3 (“almost every day”), and a cutoff score ≥ 10 corresponds to 100% sensitivity and 98% of specificity;
  - Posttraumatic Stress Disorder Checklist for DSM-5 (PCL-5): a self-report instrument used to assess symptoms of posttraumatic stress disorder using the criteria established by the DSM-5. The short version (eight items) translated, adapted, and psychometrically assessed by Osório et al. (17) and Pereira-Lima et al. (18) was used. A cutoff point ≥ 14 corresponds to sensitivity equal to 0.97 and specificity equal to 0.61.
  - Insomnia Severity Index (ISI): a 7-item self-report instrument rated on a 5-point Likert scale intended to assess the severity of insomnia in the last 2 weeks. It was adapted and validated in Brazil by Castro (19), with a cutoff point ≥ 8, sensitivity of 73%, and specificity of 80% to detect positive and negative cases of chronic insomnia.
  - Abbreviated Maslach Burnout Inventory – Human Services Survey (aMBI-HSS). It assesses the burnout syndrome based on the following dimensions: emotional exhaustion, depersonalization, and professional achievement. This self-report instrument was developed by Maslach et al. (20) and later adapted and validated in Brazil by Carlotto and Câmara (21). Its short version, proposed for and validated among health workers (22), was adopted in this study, in which a cutoff point ≥ 9 indicates emotional exhaustion, ≥ 6 indicates despersonalization, and ≥ 10 professional accomplishment.

### Procedures

Data were collected and managed using REDCap (Research Electronic Data Capture). The participants were granted access to the survey through an electronic link generated by the SURVEY application. Data were collected at seven different points in time, with a 15-day interval (Baseline, D15, D30, D45, D60, D75, and D90). A total of 1,522 participants accessed the platform, and all those who concluded the baseline assessment (*n* = 916) received the links to assess all the follow-up phases, regardless of whether they had answered the previous stages or not. The participants answered a questionnaire at the baseline to characterize the sample and the instruments intended to assess mental health. Only the instruments intended to measure mental health outcomes were completed in the follow-up. In this stage, even if the participants had not completely answered all the instruments, having completed at least one of the instruments ensured their participation in the follow-up.

### Data Analysis

Data were statistically analyzed using Statistical Package for Social Science (SPSS), version 23.0 (IBM, 2015). The responses to the different outcome instruments were dichotomized according to the cut-off points established by the aforementioned psychometric studies. Descriptive statistics were performed, and a non-parametric test (Chi-square) was used to compare the frequencies above the instruments' cutoff points obtained at the baseline and each of the follow-up stages. Binary logistic regression analyses were performed to assess potential risk and protective factors for mental health outcomes and potential survival bias. The independent variables were presented together with the description of the socio-demographic questionnaire (socio-demographic, occupational, health conditions, and perception of support and risk associated with COVID-19). For the assessment of survival bias, the outcome variables were included in the regression analysis. Odds ratios are presented with a 95% confidential interval. No methods were used to impute missing data. All the statistical tests were conducted at a 0.05 significance level.

## Results

The initial sample was composed of 916 participants from different Brazilian states/regions (Southeast region predominate). The study's remaining phases presented 55.7–22.8% response rates (D15: *N* = 510; D30: *N* = 401; D45: *N* = 319; D60: *N* = 284; D75: *N* = 240; D90: *N* = 209). The socio-demographic, occupational, and clinical characterization of the participants included in the baseline and final follow-up are presented in Table 1.

**Table 1:** Socio-demographic, clinical, and occupational characterization and participants' risk perception at the baseline and final follow-up.

**Variables**	**Respondents** ***N*** **(%)**		**Crude OR (95%CI) (*P*)**	**Adjusted OR^#^ (95%CI) (*P*)**
	**Baseline** ***N* = 916**	**Completed all the surveys (7)** ***N* = 201**		
**Gender**				
Female	730 (79.7)	160 (79.6)	0.993 (0.673–1.464) (0.97)	
Male	186 (20.3)	41 (20.4)		
**Age - mean (CI 95%)**	35.2 (34.1–38.3)	38.4 (37–39.7)	1.002 (0.998–1.007) (0.32)	
**Marital status**				
Single	482 (52.6)	99 (49.3)	1.189 (0.869–1.626) (0.28)	
Stable union	434 (47.4)	102 (50.7)		
**Lives alone**				
Yes	159 (17.4)	31 (15.7)	1.114 (0.725–1.712) (0.62)	
No	757 (82.6)	167 (84.3)		
**Religion**				
Yes	750 (81.9)	164 (81.6)	0.976 (0.651–1.462) (0.91)	
No	166 (18.1)	37 (18.4)		
**Smoker**				
Yes	94 (10.5)	19 (9.5)	1.123 (0.661–1.91) (0.67)	
No	822 (89.7)	182 (90.5)		
**Alcohol abuse**				
Yes	121 (13.2)	26 (12.9)	1.031 (0.648–1.642) (0.90)	
No	795 (86.8)	175 (87.1)		
**Drug use** ***N***				
Yes	38 (4.3)	7 (3.5)	1.298 (0.564–2.987) (0.53)	
No	877 (95.7)	194 (96.5)		
**Physical exercise**				
Yes	408 (44.5)	86 (42.8)	0.913 (0.666–1.252) (0.57)	
No	508 (55.5)	115 (57.2)		
**Previous psychiatric care**				
Yes	152 (16.6)	54 (26.9)	0.432 (0.296–0.631) (<0.001)^*^	0.619 (0.372–1.030) (0.07)+
No	764 (83.4)	147 (73.1)		
**Psychiatric diagnosis**				
Yes	198 (21.6)	67 (33.3)	0.474 (0.335–0.671) (<0.001)^*^	0.760 (0.471–1.225) (0.26)
No	718 (78.4)	134 (66.7)		
**Physical illness**				
Yes	269 (29.4)	81 (40.3)	0.528 (0.381–0.733) (<0.001)^*^	0.702 (0.471–1.047) (0.08)+
No	647 (70.6)	120 (59.7)		
**Medication use**				
Yes	393 (42.9)	109 (54.2)	0.556 (0.406–0.762) (<0.001)^*^	0.847 (0.566–1.266) (0.42)
No	523 (57.1)	92 (45.8)		
**Occupation**				
Nurse	376 (41.0)	73 (36.3)	0.775 (0.561–1.072) (0.12)	
Other (Ω)	540 (59.0)	128 (63.7)		
**Professional experience mean (CI 95%)**	10.2 (9.6–10.7)	13.2 (11.8–14.5)	1.049 (1.031–1.067) (<0.001)^*^	1.041 (1.023–1.060) (<0.001)^*^
**Works in COVID referral center**				
Yes	601 (65.6)	67 (33.3)	0.663 (0.472–0.930) (0.02)^*^	0.777 (0.542–1.115) (0.171)
No	315 (34.4)	134 (66.7)		
**Works in a public hospital**				
Yes	572 (62.4)	183 (91.0)	1.770 (1.045–2.995) (0.03)^*^	1.790 (1.031–3.108) (0.04)^*^
No	344 (37.6)	18 (9.0)		
**Works in a tertiary care facility**				
Yes	445 (48.6)	94 (46.8)	0.788 (0.576–1.078) (0.14)	
No	471 (51.4)	107 (53.2)		
**Work in the COVID-19 frontline**				
Yes	712 (77.7)	151 (75.1)	0.829 (0.575–1.196) (0.32)	
No	204 (22.3)	50 (24.9)		
**Previous SARS-COV-2 infection**				
Yes	131 (14.3)	19 (9.5)	1.779 (1.064–2.974) (0.03)^*^	1.435 (0.844–2.442) (0.18)
No	585 (85.7)	182 (90.5)		
**Concerns with being infected**				
Yes	728 (79.5)	163 (81.1)	0.878 (0.591–1.305) (0.52)	
No	188 (20.5)	38 (18.9)		
**Concerns with a family member being infected**				
Yes	879 (96.0)	192 (95.5)	0.869 (0.403–1.874) (0.72)	
No	37 (4.0)	9 (4.5)		
**People avoid contact**				
Yes	389 (42.5)	86 (42.8)	1.017 (0,741–1.395) (0.92}	
No	527 (57,5)	115 (57.2)		
**Satisfied with protective measures**				
Yes	400 (43.7)	85 (42.3)	0.930 (0.678–1.277) (0.66)	
No	516 (56.3)	116 (57.7)		
**Considers quitting the job**				
Yes	149 (6.3)	30 (14.9)	0.879 (0.569–1.358) (0.56)	
No	767 (93.7)	171 (85.1)		
**Positive expectation for the future**				
Yes	707 (77.2)	145 (72.1)	1.419 (0.993–2.026) (0.05)+	1.042 (0.709–1.533) (0.84)
No	209 (22.8)	56 (27.9)		
**Works longer than usual**				
Yes	460 (50.2)	96 (47.8)	0.882 (0.645–1.206) (0.43)	
No	456 (49.8)	105 (52.2)		
**Receives some support from the institution**				
Yes	160 (17.5)	29 (14.4)	0.871 (0.560–1.353) (0.54)	
No	756 (82.5)	172 (85.6)		
**Anxiety (GAD-7** **>** **10)**				
Yes	397 (43.3)	91 (45.3)	1.106 (0.807–1.515) (0.53)	
No	519 (56.7)	110 (54.7)		
**Depression (PHQ-9** **>10)**				
Yes	368 (40.2)	80 (39.8)	0.980 (0.712–0.349) (0.90)	
No	548 (59.8)	121 (60.2)		
**Emotional exhaustion (aMBI exhaustion** **>** **8)**				
Yes	336 (36.7)	82 (40.8)	1.251 (0.908–1.723) (0.17)	
No	580 (63.3)	119 (59.2)		
**Depersonalization (aMBI depersonalization** **>** **5)**				
Yes	167 (18.2)	33 (16.4)	0.852 (0.561–1.293) (0.45)	
No	749 (81.8)	168 (83.3)		
**Professional achievement (aMBI perso.accomp**. **>** **9)**				
Yes	760 (83.0)	164 (81.6)	0.885 (0.589–1.330) (0.56)	
No	156 (17.0)	37 (18.4)		
**Post-traumatic stress (PCL-5** **>** **13)**				
Yes	330 (36.0)	76 (37.8)	1.103 (0.798–1.525) (0.55)	
No	586 (64.0)	125 (62.2)		
**Insomnia (ISI** **>** **7)**				
Yes	563 (61.5)	123 (61.2)	0.986 (0.715–1.359) (0.93)	
No	356 (38.5)	78 (38.8)		

# *Adjusted for variables with significant crude OR p (< 0.05) or with a tendency toward significance (p < 0.1)*.

* *Significant; ^+^tendency toward significance (p < 0.1)*.

*Ω: Other = Baseline: 30% physicians; 29% other professions (11.4% physical therapists, 6.2% psychologists, 3.1% nutritionists, 2.8% pharmacists, 2.0% speech therapists, 1.7% social workers, 1.1% dentists, 0.7% were occupational therapists); All surveys: 31.3% physicians; 32.4% other professions (11.9% physical therapists, 6.5% psychologists, 5.0% nutritionists, 2.0% pharmacists, 3.5% speech therapists, 1.0% social workers, 1.5% dentists, 1% were occupational therapists)*.

Table 1 shows that the groups differed significantly regarding previous psychiatric care, psychiatric diagnosis, physical illness, medication use, years of professional experience, whether they worked in a COVID-19 referral center, worked in a public hospital, had previously been infected with SARS-COV-2, and whether they held positive professional prospects. Considering the high rate of loss to follow-up, we checked whether there was potential survival bias among the participants, impacting the results. Adjusted logistic regression analyses indicated that having more years of experience (OR = 1.04; CI 95%: 1.02–1.06; *p* < 0.001) and working in a public hospital (OR = 1.79 CI 95%: 1.03–3.11; *p* = 0.04) positively impacted whether the participants remained in the study. Note that the sample that completed the study does not differ from the initial sample regarding the initial outcome measures; that is, these variables did not impact survival.

Data concerning the progression of emotional exhaustion indicators throughout the follow-up are presented in Table 2.

**Table 2:** Follow-up of mental health indicators amog Brazilian health workers providing care to COVID-19 patients.

**Measures**	**Days of follow-up**						
	**Baseline**	**15**	**30**	**45**	**60**	**75**	**90**
**Anxiety**							
Number of respondentes	916	510	401	319	284	240	209
% of GAD 7 ≥ 10	43.3	36.5^*^	31.7^*^	32.9^*^	29.2^*^	30.8^*^	30.6^*^
**Depression**							
Number of respondentes	916	501	399	315	277	277	205
% of PQH 9 > 10	40.2	36.7	34.8	37.1	37.2	33.1^*^	35.1
**Insonia**							
Number of respondentes	916	485	391	304	271	229	201
% of ISI ≥ 8	61.5	59.0	55.5^*^	54.3^*^	56.5	51.5^*^	59.2
**Posttraumatic stress disorder**							
Number of respondents	916	488	392	305	271	230	201
% of aPCL 5 > 13	36.0	35.7	32.7	34.8	32.1	32.2	35.3
**Burnout (emotional exaustion)**							
Number of respondents	916	495	395	310	273	234	202
% of AMBI-EE > 8	36.6	42.28^*^	41.3	38.7	42.9	39.7	8.1
**Burnout (depersonalization)**							
Number of respondents	916	495	396	311	273	235	202
% of AMBI-D > 5	18.2	20.2	20.7	20.3	19.8	24.7^*^	22.3
**Bunout (professional achiviement)**							
Number of respondents	916	495	395	310	273	235	202
% of AMBI-PA >9	83.0	71.5^*^	74.7^*^	71.4^*^	68.9^*^	65.5^*^	67.3^*^

* *The difference between the baseline and follow-up day was statistically significant (Chi-square – p < 0.05)*.

High rates were found throughout the study for all the indicators of mental distress (except despersonalization) at all stages of data collection (>30%), especially insomnia (>51%). However, anxiety indicators were the only ones that presented a statistically significant decrease in all the phases compared to baseline.

Depression and insomnia symptoms showed a significant drop in isolated points of the assessment, which were not maintained at the final follow-up. Posttraumatic stress indicators remained stable throughout the study, as did Burnout indicators related to emotional exhaustion and depersonalization, 40 and 20%, respectively. In turn, professional achievement significantly decreased by approximately 19%.

Logistic regression analyses were performed to identify potential risk and protective factors associated with indicators of emotional overload at the end of the follow-up. The multicollinearity analysis shows coefficients of tolerance >0.1 (0.30–0.93) and VIF < 10 (1.08–4.4), suggesting that the independent variables are not correlated. Furthermore, the Hosmer-Lemeshow goodness of fit test for the set of independent variables indicates that the model was adequate. Occupational and personal characteristics, such as profession (other health professions: OR = 3.26; IC 95%: 1.31–8.09; *p* = 0.01), lack of positive professional prospects (OR = 2.16; IC 95%: 0.86–5.42; *p* = 0.10), and religion (OR = 3.70; IC 95%: 1.13–12.05; *p* = 0.03) were associated with a greater likelihood of decreased anxiety symptoms. In addition, conditions concerning the organizational contexts were considered risk factors for the professional achievement outcome: working in a private hospital (OR = 7.30; IC 95%: 2.12–25.19; *p* = 0.002), in a secondary care facility (OR = 7.30; IC 95%: 2.12–25.19; *p* = 0.002), and being dissatisfied with physical health-protective measures (OR = 3.10; IC 95%: 1.27–7.57; *p* = 0.01). Being a physician or a nursing staff member appeared as a risk factor preventing professional achievement though it was not significant in the adjusted OR. These findings are presented in Table 3.

**Table 3:** Characteristics at the baseline associated with a decreased number of participants with anxiety (GAD 7>10).

**Characteristics**	**Change in anxiety diagnosis** **(GAD** **>** **10)** ***N*** **(%)**			**Crude OR** **(IC-95%) (*p*)**	**Adjusted OR^#^** **(IC-95%) (*p*)**
	**Baseline +**	**Baseline +**	**Total**		
	**End point +**	**End point –**			
**Occupation**					
Other health professions	19 (35.8)	34 (64.2)	53 (100)	2.526 (1.094–5.837) (0.03)^*^	3.260 (1.313–8.091) (0.01)^*^
Nurse	24 (58.5)	17 (41.5)	41 (100)		
**Religion**					
Yes	32 (41.6)	45 (58.4)	77 (100)	2.578 (0.864–7.692) (0.09)+	3.697 (1.134–12.054) (0.03)^*^
No	11 (64.7)	06 (35.3)	17 (100)		
**Positive expectations for the future**					
No	25 (39.7)	38 (60.3)	63 (100)	2.105 (0.878–5.043) (0.09)+	2.156 (0.857–5.421) (0.10) +
Yes	18 (58.1)	13 (41.9)	31 (100)		
**Characteristics**	**Change in professional achievement** **(aMBI perso.accomp > 10)s** ***N* (%)**			**Crude OR** **(IC-95%) (*p*)**	**Adjusted OR^#^** **(IC-95%) (** ***p*** **)**
	**Baseline** **+**	**Baseline** **+**	**Total**		
	**End point** **+**	**End point –**			
**Occupation**					
Other health professions	91 (82.0)	20 (18.0)	111 (100)	1.916 (0.897–4.091) (0.09)+	1.930 (0.838–4.445) (0.12)
Nurse	38 (70.4)	16 (29.6)	54 (100)		
Other health professions	85 (73.9)	30 (26.1)	115 (100)	2.588 (1.002–6.686) (0.05)+	1.931 (0.643–5.797) (0.24)
Doctor	44 (88.0)	6 (12.0)	50 (100)		
**Type of hospital**					
Private hospital	7 (46.7)	8 (53.3)	15 (100)	4.980 (1.667–14.876) (0.004)^*^	7.302 (2.117–25.189) {0.002)^*^
Public hospital	122 (81.3)	28 (18.7)	150 (100)		
**Type of care**					
Secondary care	54 (72.0)	21 (28.0)	75 (100)	4.980 (1.667–14.876) (0.004)^*^	7.302 (2.117–25.189) {0.002)^*^
Tertiary care	75 (83.3)	15 (16.7)	90 (100)		
**Satisfaction with protective measures**					
No	63 (70.8)	26 (29.2)	89 (100)	2.724 (1.215–6.104) (0.02)^*^	3.104 (1.273–7.566) (0.01)^*^
Yes	66 (86.8)	10 (13.2)	76 (100)		

# *Adjusted for variables with significant crude OR p (< 0.05) or with a tendency toward significance (p < 0.1)*.

* *Significant; ^+^tendency toward significance (p < 0.1)*.

## Discussion

To the best of our knowledge, this is the first study analyzing the progression of mental health indicators among Brazilian health professionals providing care to COVID-19 patients. There was a considerable loss to follow-up (78%); however, this rate is in line with those reported by similar studies such as Czeisler et al. (23) in the United States (76.8%) and Fancourt et al. (24) in the United Kingdom (89.9%). These studies addressed the general population and possibly portray the peculiarities of studies adopting online surveys in the pandemic context.

Nevertheless, comparisons of the our samples at the beginning of the study and end of the follow-up did not show significant differences regarding the socio-demographic variables (e.g., age, sex, marital status, and profession) and interest variables (baseline mental health and burnout indicators), suggesting that the results are comparable without the presence of bias in participant retention. This aspect deserves attention and should be highlighted as a differential of the study, since in follow-up studies in the area of mental health before (25, 26) or during the pandemic (23) biases in relation to demographic aspects were commonly portrayed.

For example, in Lamers et al. (26) and Czeisler et al. (23) greater loss to follow-up was observed when participants were younger and less educated, as well as in relation to the participants' previous mental conditions (greater loss to follow-up among participants with greater depressive and/or anxious symptoms (23, 27, 28). These factors may favor a bias in the reading of the data, with more optimistic interpretations of the results. In different longitudinal studies carried out in countries such as China (29–31), Belgium (32), Argentina (10), Netherlands (33) and Singapore (34), to assess the progression of mental health indicators among workers during pandemic, controversial results are reported, and the authors rarely pay attention to this aspect, which may be one of the factors that explain such divergences.

Concerning the study's primary objective, our findings indicate that depression, post-traumatic stress, and insomnia indicators remain high compared to studies conducted before the pandemic in Brazil (35, 36). On the other hand, the results also indicate that anxiety symptoms decreased during the follow-up, suggesting that workers are less apprehensive with the COVID-19 context than at the beginning of the pandemic or due to attenuating the number of new cases as the end of the first wave approaches. Despite this, professional accomplishment remained lower than baseline throughout the follow-up, suggesting dissatisfaction with working conditions.

Studies conducted in Europe and Asia indicate a general tendency toward decreased mental distress symptoms (e.g., anxiety, depression, impact of adverse events, perceived stress, stigma, and somatization) in specific populations such as nurses, resident physicians, and other health workers (29, 30, 32–34). Institutional factors were accounted for decreased symptoms in these countries because health managers quickly organized and arranged more beds and field hospitals, implemented rotation schedules to enable workers to rest, and provided protective equipment, among others, which decreased pressure on the health system and improved the quality of working conditions (34). This drop in the indicators was also associated with strengthening the professionals' coping strategies as they often received emotional support to adapt to the pandemic more competently, which was gradually controlled with social isolation measures (29). In addition, the governments of some countries provided financial support and cared for the workers' families, which may have contributed to alleviating their concerns (29, 34). Stigma, initially experienced by health workers (37, 38), also subsided through the media and community actions intended to sensitize the population regarding these professionals' contributions during the pandemic (34).

On the other hand, a study conducted in Argentina, a Latin American country with social and economic conditions and pandemic indicators similar to those in the Brazilian context, reported an increase in common mental disorders and decreased perceived performance (10). This result, coupled with this study's finding that professional achievement decreased, suggests that, in addition to institutional peculiarities, the various world realities, with different resources for management and investment in health actions, can have a different impact on the mental health of health professionals and on professional achievement/satisfaction. According to Freitas et al. (7), some countries such as Brazil, with high levels of social inequalities and low investment in public and health policies, suffer the impact of the pandemic in a more pronounced way compared to more developed countries, with an impact on different levels. Thus, there seems to be a greater fragility regarding the mental health of health professionals from low- and middle-income countries, which should be explored and better understood in future studies that have this specific objective. In addition, it should be noted the need to invest in care, assistance and support actions for health professionals to better cope with the pandemic in these realities, as occurred in countries such as China (29).

Despite this, it is noteworthy that a study carried out in the Netherlands (33) found an increase in the rates of burnout indicators (about 13%) comparing the onset of the pandemic and the period of greater control of the same in that country (December 2019 and June 2020). Hence, even in countries with more resources, fighting the pandemic led to feelings of hopelessness, lack of control, and inability, decreasing professional achievement, engagement in activities (depersonalization), and favoring exhaustion and moral distress (33). This fact may favor an increase of risk factors against physical and mental health, commonly associated with burnout, including the consumption of alcohol, isolation, risk of suicide, poor self-care, and medical errors (39, 40).

The fact that the rates of mental problems in our sample are still possibly high compared to the pre-pandemic period, suggests that the pandemic has been eroding the workforce in a worrying way. This is also the case in other countries, including those where a reduction in indicators has been observed (2, 3, 41, 42), indicating an important public health problem and considerable risks for work performance and patient safety (43). This is because previous studies point to an important association between the presence of burnout, cognitive dysfunction, professional performance, medical errors, client dissatisfaction and inadequate preparation for the response to COVID-19 (44–47).

In addition, previous studies conducted during the SARS (Severe acute respiratory syndrome) outbreak report persistent psychiatric symptoms among health workers from 1 to 3 years later, indicating the future is uncertain and responses to the psychological stress caused by the pandemic may change anytime depending on the context (34), so that, the need for constant monitoring and identification of risk and protective factors in mental health is essential.

In our study, the type of profession stood out as protective factors, especially for the reduction of anxiety symptoms. Professionals in fields other than nursing fields show greater chances of having reduced anxiety experiences, a fact that in cross-sectional studies has been attributed to the condition of these professionals having less and less costly contact with the patient, not always acting in the line of front (5). Nevertheless, the longitudinal study conducted by Lui et al. (29) reports that nursing workers presented the most significant improvement in mental health indicators; this group obtained the worst scores at the beginning of the pandemic. Various governmental actions supporting the fight against the pandemic were accounted for such improvement. Factors such as religion also stood out as a social determinant of health (48–50). A curious fact is that workers presenting less positive expectations regarding their professional future were more likely to experience decreased anxiety. Such finding is possibly explained by the fact that demotivated workers, or those experiencing high burnout levels even before the pandemic or with negative professional prospects, were less intensively impacted by the pandemic, reflecting defense mechanisms based on conformism and avoidance (51).

Risk factors for burnout/professional frustration include the workplace (private hospital and secondary care facility) and dissatisfaction with physical protective measures, possibly emphasizing the role of the specific conditions of the settings where the services are provided, such as workers having received training to care for patients requiring less complex care and lack of equipment required in emergencies, among others.

International studies report individual variables such as previous history of stress (32), being concerned with potential infection (10) and the pandemic repercussions (10, 30), living alone, especially during social isolation (34), and perception of not having been adequately qualified/trained for the job (30), were the main risk factors reported by some longitudinal studies addressing mental health problems.

The findings show that the indicators of emotional distress of professionals are high, and that only the symptoms of anxiety decreased in the different phases of the study, in relation to the baseline. On the other hand, the professional fulfillment of around 19% of the participants also declined over the course of the pandemic. The data portray the mental health condition of Brazilian health professionals who provide care to patients with COVID-19, but they need to be viewed and generalized with caution, given some possible limitations of the study: (a) sample loss (which may be associated with several factors, including the long length of the data protocol and the substantial number of reassessments); (b) the lack of control over the different sources of recruitment and the participant's professional status (since proof of work in the health area and with patients with COVID-19 was not required for inclusion in the study); (c) data collection methodology (online recruitment, self-report instruments); (d) lack of control over participants' pre-pandemic mental health measures; (e) the fact that Brazil is a continent-spanning country that presents peculiar characteristics regarding the evolution of the pandemic.

## Conclusion

Even though the results indicate that anxiety indicators among health workers decreased through the first wave pandemic, the remaining indicators remain high compared to parameters from before the pandemic. On the other hand, professional overload seems to be on the rise, which requires constant attention and monitoring, in addition to measures intended to minimize impacts on individuals and the collective. We can hypothesize that socioeconomic differences between countries could impact workers' mental health care to COVID-19 patients. They directly influence working conditions, which can act as protective or risk factors for a better response to the emotional impacts associated with the COVID-19 pandemic. Future studies that more specifically explore the impact of these variables are opportune.

## Data Availability Statement

The raw data supporting the conclusions of this article will be made available by the authors, without undue reservation.

## Ethics Statement

The studies involving human participants were reviewed and approved by Comitê de Ética em Pesquisa com Seres Humanos do Hospital das Clínicas da Faculdade de Medicina de Ribeirão Preto – USP. The patients/participants provided their written informed consent to participate in this study.

## Author Contributions

FO, AZ, JC, JH, KP-L, and SL: conception and design and substantial contributions to drafting the article or revising it critically for important intellectual content. FO, IS, AZ, and SL: collect, analysis, and interpretation of data. FO, AZ, IS, JC, JH, KP-L, and SL: final approval of the version to be published. All authors contributed to the article and approved the submitted version.

## Funding

This work was supported by Ministry of Health of Brazil / National Council for Scientific and Technological Development (Cnpq – Process No. 401058/2020-4; Cnpq – Process No. 465458/2014-9; Productivity Research Fellows: no. 302601/2019-8 (FO); 307945/2018-9 (SL); The São Paulo Research Foundation No. 2014/50891-1). The funders had no role in this study' design, analysis, interpretation, or publication.

## Conflict of Interest

The authors declare that the research was conducted in the absence of any commercial or financial relationships that could be construed as a potential conflict of interest. The reviewers SP and Y-PW declared a shared affiliation with the authors FO, AZ, IS, JC, JH, KP-L, and SL at the time of review.

## Publisher's Note

All claims expressed in this article are solely those of the authors and do not necessarily represent those of their affiliated organizations, or those of the publisher, the editors and the reviewers. Any product that may be evaluated in this article, or claim that may be made by its manufacturer, is not guaranteed or endorsed by the publisher.
